# Why a collapse of global civilization will be avoided: a comment on Ehrlich & Ehrlich

**DOI:** 10.1098/rspb.2013.1193

**Published:** 2013-09-22

**Authors:** Michael J. Kelly

**Affiliations:** Department of Engineering, University of Cambridge, Cambridge CB3 0FA, UK

Ehrlich FRS & Ehrlich [1] claim that over-population, over-consumption and the future climate mean that ‘preventing a global collapse of civilization is perhaps *the* foremost challenge confronting humanity’. What is missing from the well-referenced perspective of the potential downsides for the future of humanity is any balancing assessment of the progress being made on these three challenges (and the many others they cite by way of detail) that suggests that the problems *are* being dealt with in a way that will *not* require a major disruption to the human condition or society. Earlier dire predictions have been made in the same mode by Malthus FRS [2] on food security, Jevons FRS [3] on coal exhaustion, King FRS & Murray [4] on peak oil, and by many others. They have all been overcome by the exercise of human ingenuity just as the doom was being prophesied with the deployment of steam engines to greatly improve agricultural efficiency, and the discoveries of oil and of fracking oil and gas, respectively, for the three examples given. It is incumbent on those who would continue to predict gloom to learn from history and make a comprehensive review of human progress before coming to their conclusions. The problems as perceived today by Ehrlich FRS and Ehrlich will be similarly seen off by work in progress by scientists and engineers. My comment is intended to summarize and reference the potential upsides being produced by today's human ingenuity, and I leave the reader to weigh the balance for the future, taking into account the lessons of recent history.

The population explosion (and its Malthusian societal disruptions) that Ehrlich FRS predicted for the 1990s has not come about [5,6], and the concerns in this present Ehrlich paper are not tempered by the mounting evidence of the demographic transition that occurs when the majority of people live in cities and have access to education. In Japan, Europe and North America the population, excluding immigration, is in decline. Some studies indicate that a peak of 9 billion people in 2050 will be followed by a decline to a population of approximately 6 billion in 2100—less than that in 2000 [7] and bringing new problems of unwanted infrastructure assets! The UN is revising its future population estimates downward [8]. If we look at the waste in the contemporary food chain, at the point of growth, in transit to the market and into the homes of consumers, and compound that loss by the amount of food thrown out rather than consumed, we generate the quantity of food to feed the 9 billion today with the systems in place if we were less wasteful and could distribute it [9].

Animal protein is now being generated in the laboratory and not on the farm [10]. Where is the discussion of the impact of mega-cities being self-sufficient in animal protein from factories within their city boundaries 40 years from now? This is the time scale on which synthetic fibre comprehensively displaced wool from most of its markets. Indeed, rather than speak of peak oil, we can speak of peak farmland—we will need smaller areas in future to feed the world, and we will oversee the managed return of excess land to the wild [11].

The starkest example in the consideration of material overconsumption is the smart phone [12]. This was developed within the paradigm of business as usual to improve the way in which we communicate. Two points are relevant. First, the small piece of metal, plastic and semiconductor that fits in the palm of a hand contains the functions of a camera, radio, telephone, answering machine, photo album, dictaphone, music centre, satellite navigation system, video camera and player, compass, stop-watch, Filofax, diary and more, which were all separate and bulky items only 20 years ago. This represents the great dematerialization of modern civilization, well ahead of any imminent collapse of natural resources. The shape of high streets and retail centres are changing to reflect this evolution. Indeed, the recycling of electronic systems will enhance further this capability of doing more with less material, and the market for extended time between recharging has driven extraordinary improvements in energy efficiency. It is these new low-resource technologies with ever-increasing recycled materials that will drive the world in future. Second, the mobile phone is being used in rural Africa and India to inform farmers of optimal times for taking their products to market, thus reducing greatly the loss of product and/or income, and reducing the stress on land from the need to overproduce to compensate for such losses [13]. Peak planet is now the new research topic [14].

Any perceived threat to the security of the energy supply from finite resources over the last 200 years has been met by a deeper search for reserves. Hansen *et al.* [15], and especially their fig. 6, show just how little (approx. 10%) of the known and accessible fossil fuel reserves (both conventional and unconventional) has been consumed, and we have had 40 years of future energy reserves to hand for some time [16]. We have not stopped looking for more, as with the recent discoveries of huge fields of methyl hydrates. In future, when we leave the fossil fuel age, it will not be because of the exhaustion of fossil fuels, but because a cheaper, cleaner and more convenient alternative technology emerges, and we have ample time, probably 100 years, to get there.

Modern climate scientists seem to be fixated on human-produced CO_2_, and have missed what the Sun [17] and the biosphere [18] have been doing for the last 30 years. *If* the history of solar behaviour repeats itself and we were to enter another little ice age, every ppm of CO_2_ in the atmosphere would be a boon as we feed 9 billion people in 2050 compared with the less than 1 billion last time in the seventeenth to eighteenth centuries. The transition out of the Medieval Warm Period into the Little Ice Age harmed but did not collapse global civilization, and we are much better prepared this time. The growing amplitude of the Keeling cycles of CO_2_ in the atmosphere is evidence of the greening of the biosphere [18]. The present temperature stasis since 1998, if extended by another 5 years, as now suggested [19] at a time of ever-increasing CO_2_ emissions, implies that both the coupling between CO_2_ and globally averaged surface temperatures has been exaggerated in the climate models and natural variability has been underestimated. Indeed, Otto *et al*. [20] have just revised down their estimate of climate sensitivity to atmospheric CO_2_ to a value that is now half that cited in earlier IPCC reports. Akasofu's [21] projection of the future temperature, made originally in 2000, and based on extending previous climatic cycles without explicit reference to CO_2_, has been borne out very precisely, and it is more accurate than all the climate model projections put together—furthermore, he makes a projection of lower temperatures until 2030!

An over-emphasis on the urgency of mitigation has had a direct societal consequence in the Gadarene rush to reduce fossil fuel consumption. We do have more time to develop proper alternatives to fossil fuels. The current bankruptcies of alternative energy companies are inevitable: their present technology is both immature and uncompetitive. It is an exact repeat of what happened in California in the 1980s in response to the 1970s oil crisis and for the same reasons: without massive subsidy the energy generated did not produce the profits needed to keep up maintenance. (Graphic images of green industrial dereliction can be seen by googling the phrases ‘abandoned solar farms’ and/or ‘abandoned wind farms’.) Two hundred years ago, windmills stopped turning with the advent of steam engines, which were more efficient, needed less maintenance, and provided energy when and where needed. Little has changed in relative terms since! Trends in solar photovoltaics suggest that in 20 years the technology could become absolutely competitive with fossil fuels [22] unless the price of the latter collapses from current high prices just as they did after the 1970s peak. Whatever happens, the total energy from practical and economic solar systems will play a small part in meeting the global energy demand for the foreseeable future: renewable energy sources are intrinsically dilute at source [23]. Energy storage at the large scale is way into the future, except for water for hydroelectricity, as in New Zealand and Norway. Pushing water uphill with alternative energies is woefully inefficient.

Communications, new materials and health systems all present humanity with clear opportunities to avoid future problems with tools not available to earlier generations. The Internet, and its implication of all information available everywhere, instantaneously for everyone, will ensure that technical, medical and societal advances will proceed and propagate very rapidly. An advance in one corner of the world will almost instantaneously be accessible and adaptable anywhere. Human travel will change from becoming a necessity to an option, freeing up time, reducing emissions and enhancing business between continents [24]. New ‘designer’ materials and three-dimensional printing technology for manufacture are likely to massively reduce our reliance on depleting natural resources, providing for a far more adaptive approach to materials in applications. The incredible waste we currently produce is likely to reduce very significantly, making for greater resilience against resource depletion [25]. Ehrlich & Ehrlich [1] are concerned about future pandemics in a closely interconnected world. However, advances in medicine and diagnostics will result in significant economic gains in terms of treatment efficacy, in days lost from the workplace and in the ability of mankind to respond to a future pandemic. The recent response to the H5Nn series of bird flu viruses is very encouraging, and the strategies have existed for some time [26]. We can be a much more resilient race in future than we could be in the past. Similarly, with the advances in understanding the brain and President Obama's recent commitment to mapping the brain, we will enhance our cognitive and processing capability so as to further our ingenuity and resilience in response to future threats.

The mainstream scientific and engineering community can see nothing that suggests an imminent collapse of civilization, and it is well on track to deal with new problems as they emerge, in continuity with the history of the last 200 years. Neo-Malthusians have proved comprehensively wrong so far, and this comment argues that this is set to continue into the foreseeable future. This comment is not denying challenges, but is really questioning defeatism. Weigh the evidence.

Finally, it is only civilizations backed by strong economies that are in a position to do the research and make the necessary scientific, engineering and technological advances to offset environmental threats. Scientific views that undermine economic progress are a threat in themselves, and need a careful and robust justification before they are widely propagated.
